# The Role of B Cells in Adult and Paediatric Liver Injury

**DOI:** 10.3389/fimmu.2021.729143

**Published:** 2021-09-23

**Authors:** Arzoo M. Patel, Yuxin S. Liu, Scott P. Davies, Rachel M. Brown, Deirdre A. Kelly, Dagmar Scheel-Toellner, Gary M. Reynolds, Zania Stamataki

**Affiliations:** ^1^ Centre for Liver and Gastrointestinal Research, Institute of Immunology and Immunotherapy, University of Birmingham, Birmingham, United Kingdom; ^2^ Institute of Inflammation and Ageing, University of Birmingham, Birmingham, United Kingdom; ^3^ Department of Histopathology, Queen Elizabeth Hospital, Birmingham Women’s and Children’s National Health Service (NHS) Foundation Trust, Birmingham, United Kingdom; ^4^ The Liver Unit, Birmingham Women’s and Children’s Hospital and the University of Birmingham, Birmingham, United Kingdom

**Keywords:** B cell, liver, liver fibrosis, biliary atresia, paediatric liver disease, liver diseases

## Abstract

B lymphocytes are multitasking cells that direct the immune response by producing pro- or anti-inflammatory cytokines, by presenting processed antigen for T cell activation and co-stimulation, and by turning into antibody-secreting cells. These functions are important to control infection in the liver but can also exacerbate tissue damage and fibrosis as part of persistent inflammation that can lead to end stage disease requiring a transplant. In transplantation, immunosuppression increases the incidence of lymphoma and often this is of B cell origin. In this review we bring together information on liver B cell biology from different liver diseases, including alcohol-related and metabolic fatty liver disease, autoimmune hepatitis, primary biliary and primary sclerosing cholangitis, viral hepatitis and, in infants, biliary atresia. We also discuss the impact of B cell depletion therapy in the liver setting. Taken together, our analysis shows that B cells are important in the pathogenesis of liver diseases and that further research is necessary to fully characterise the human liver B cell compartment.

## Introduction

Liver disease is responsible for approximately 3.5% of deaths worldwide, with liver cirrhosis being the 11^th^ most common cause of morbidity (1). As a consequence, there is high demand for donor livers for transplantation, the only effective current treatment. This makes the liver the second most frequent solid organ transplanted, with less than 10% of liver transplant needs being met (1); the discovery of alternative treatments is therefore essential in reducing the global demand for donor livers. In recent years, therapies which manipulate the immune system, an underlying factor in many disease settings, have reported efficacy in the liver (2). These approaches require an in-depth understanding of how cells of the adaptive immune response contribute to the progression of disease. B cells play a central role in the protection against pathogens, whilst also contributing to immune regulation and the maintenance of self-tolerance. B cells are also known to contribute to the pathogenesis of autoimmune disorders through the production of autoantibodies, antigen presentation and the secretion of pro-inflammatory cytokines (3, 4). The role of B cells in other chronic liver diseases is less clear. In this review, we will discuss descriptions of liver B cell subsets and how they may contribute to inflammation in the liver, with possibilities for therapeutic intervention.

## B Cell Development and Differentiation

**Figure 1:**
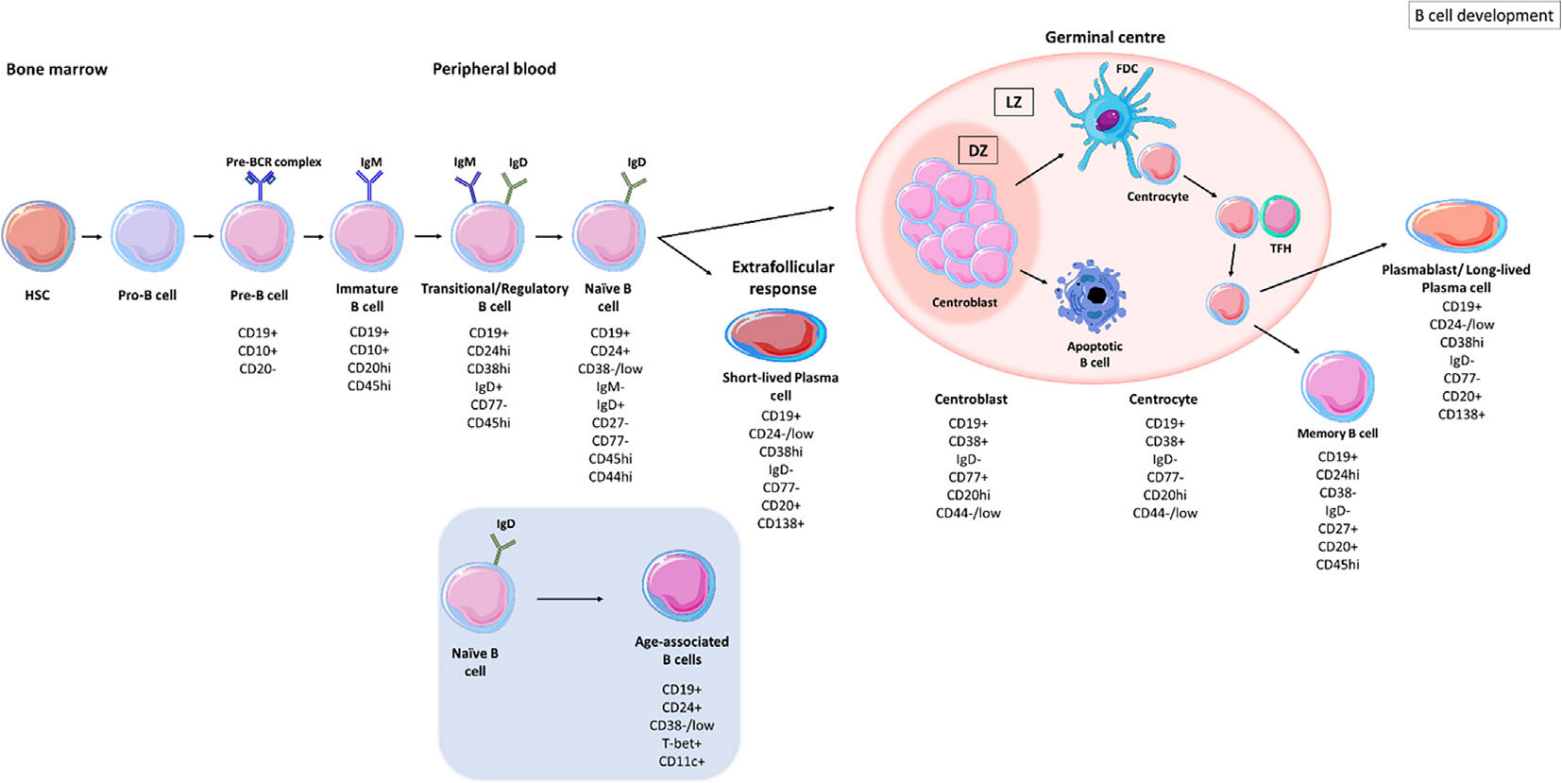
B cell development stages. B cells develop in the bone marrow from haematopoietic stem cells (HSCs), progressing from pro-B cell stages to pre-B cells before migrating into the circulation as transitional B cells. Upon antigen recognition, activated B cells migrate to secondary lymphoid organs and enter germinal centres where they undergo clonal expansion and somatic hypermutation (SHM) within the dark zones (DZ). B cells with disadvantageous mutations die by apoptosis whereas those B cells with improved receptor affinity interact with follicular dendritic cells (FDC) and T follicular helper cells (TFH), in the light zone (LZ). B cells undergo class switch recombination (CSR) and receive survival signals to differentiate into memory B cells and long-lived plasma cells (PCs) (5). Naïve B cells can differentiate into short-lived plasma cells through extrafollicular responses (6). Naïve B cells can also differentiate into age-associated B cells (ABCs) upon stimulation (7).

B cells develop from haematopoietic stem cells (HSCs) in the bone marrow and progress from pro-B cell stages (expressing CD45 isoform B220) to pre-B cell stages (expressing CD19) (**Figure 1**) (8). The formation of the pre-B cell receptor (pre-BCR) involves the rearrangement and assembly of heavy and light immunoglobulins chains (8). B cells that possess a non-functional BCR are then deleted and those with an autoreactive pre-BCR either undergo apoptosis or receptor editing to produce a functional BCR (8, 9). These B cells further develop into immature B cells that express immunoglobulins (Ig)M and IgD. Immature B cells undergo another checkpoint where their BCR reactivity against autoantigens is monitored; B cells with high autoreactivity or low autoreactive BCRs are either deleted or undergo receptor editing to produce a functional BCR (10). Activation-induced cytidine deaminase (AID) is important in central B cell tolerance; Meyers et al., showed that there was an increase in the frequency of autoreactive clones, exiting the bone marrow, in AID-deficient patients (11). Developing B cells from humanized mice, deficient in AID expression failed to remove autoreactive clones displaying a vital role for AID expression in central B cell tolerance (12). Immature B cells with an autoreactive BCR, expressing recombination-activating gene 2 (RAG2) undergo secondary recombination to produce a non-autoreactive BCR (12). Those B cells with non-autoreactive BCRs then exit the bone marrow into the periphery and are termed transitional B cells (13, 14).

Transitional B cells are defined as CD19+ CD24hi CD38hi CD77- and express surface IgM (sIgM) and surface IgD (sIgD) (15). CD19+ CD24hi CD38hi populations also contain regulatory B cells (B-regs) which control the immune response through interleukin (IL)-10 and transforming growth factor β (TGF-β) secretion (16, 17). Transitional B cells migrate to secondary lymphoid organs (SLO) where they mature into naïve B cells, defined by CD19+ CD27- IgM+ IgD+ (CD24+ CD38-/low) waiting to encounter an antigen (5, 18, 19). If naïve B cells do not encounter their cognate antigen, they re-circulate back into the periphery and die within several days (5).

Upon antigen recognition, naïve B cells become activated and either differentiate to IgM-producing plasma cells as part of the extrafollicular response, where they form short-lived plasma cells (6) or enter secondary lymphoid tissues where they encounter T cells in the T cell zone. B cells that are co-stimulated by T cells enter B cell follicles where they differentiate into proliferating centroblasts forming a germinal centre (GC) (**Figure 1**) (20). Centroblasts rapidly proliferate in the dark zone of the germinal centre and somatic hypermutation (SHM) enters point mutations into the variable region genes. In the light zone, now differentiated to centrocytes (21), the B cells undergo selection based on affinity of their BCR. Centrocytes sample antigen from the surface of follicular dendritic cells and present it to follicular helper T cells (TFH) to undergo selection (22). Centrocytes may regain entry to the dark zone for further receptor editing or to undergo class-switch recombination (CSR) and leave the GC as memory B cells or as precursors to long-lived plasma cells (23).

Based on their IgD, CD27, CD38 and CD24 expression B cells can be separated into subpopulations of naïve and memory B cells. Common proteins that are used to identify B cell subsets are listed in **Table 1**. Unswitched memory B cells express IgM and CD27 on their surface, classical switched memory B cells are IgD- CD27+ and IgD- CD27- B cells, referred as double-negative (38, 57–60). This cell type is increased in inflammation caused by autoimmunity (58) or infection (61). Memory B cells that encounter antigen proliferate rapidly and mount a robust immune response (62). CD19+ CD24-/low CD38hi IgD- CD77- PBs are terminally differentiated B cells capable of secreting high affinity antibodies (15). PBs leave the GC and circulate in the blood to the bone marrow or to further target organs, where they further differentiate into long-lived plasma cells (PCs) (CD138+) that receive survival signals from their niche (62–65).

**Table 1:** Common proteins that are used to differentiate B cell subsets.

Marker	Function	Reference
CD1d	May enable B cells to present antigens to invariant NKT cells Expressed in naïve and memory B cells, in plasma cells and in regulatory B cells	(24)
CD5	Negative regulator of BCR signalling Protects B cells from apoptosis after BCR stimulation Supports B cell survival *via* IL-10 production	(25)
CD10	Role in pre-B cell maturation and differentiation	(26)
CD11b	Forms part of the complement receptor 3 present on the surface of B cells	(27)
CD11c	Integrin, alpha X (complement component 3 receptor 4 subunit) (ITGAX) found on activated B cells	(27)
	Expressed on age-associated B cells	(28)
	Marks memory cells, precursors of antibody-secreting cells	(29)
CD19	Co-receptor required for BCR signal transduction	(30)
	Cooperates with CD21 for BCR-independent signalling	(31)
CD20	Pan-B cell surface marker for mature B cells Lost during terminal B cell differentiation	(32)
	Regulator of calcium flux triggered by BCR Required for optimal B cell responses to T-independent antigens	(33)
CD21	B cell co-receptor required to enhance BCR signalling, complement receptor	(34)
CD24	On activated B cells, CD24 facilitates CD4+ T cell clonal expansion *via* co-stimulation	(35)
	Role in the regulation of B cell development	(36)
CD27	Promotes the differentiation of memory B cells into plasma cells	(37)
	Marker of B cell activation/memory	(38)
CD38	Involved in B cell differentiation	(39)
	Crosslinking of CD38 to the BCR reduces the threshold for B cell activation	(40)
CD44	May play a role in antigen-dependent B cell differentiation	(41)
	Interacts with the polysaccharide hyaluronan (HA) in the extracellular matrix	(42)
CD45	Central regulator of BCR signalling	(43)
CD77	GC B cell entering apoptosis (CD77+)	(44)
	Marker of GC B lymphocytes	(45)
	Discriminator of centroblasts (CD77+) and centrocytes (CD77-)	(46)
CD80/CD86	Co-stimulatory molecules	(47, 48)
CD138	Syndecan 1, regulates the survival of plasma cells and long-term humoral immunity	(49)
FcRL4	Expressed on the surface of a subset of memory B cells	(50, 51)
	Expressed on the surface of atypical memory B cells	(52)
	Potential function in mucosal immunity	(53)
FcRL5	Expressed on the surface of atypical memory B cells	(52)
	Novel IgG receptor, inhibits BCR signalling May have a dual signalling capacity (CD21 co-engagement may result in B cell activation)	(54)
T-bet	Promotes the survival of memory B cells and IgG2a isotype switching	(55, 56)

Brief descriptions of the putative protein functions are given with associated references, but often the precise role of the proteins within B cell subpopulations may not be clear.

B cells with an exhausted memory-like phenotype are expanded in the peripheral blood of the elderly and are termed age-associated B cells (ABCs) (60). ABCs are characterised as CD19+ CD21lo CD11b+ CD11c+ and express the transcription factor, T-bet (60, 66–68). CD21 low B cell populations are likely to be heterogeneous and can show distinct stages of differentiation in different diseases. In SLE, they have been described as antibody secreting cells with germline-encoded Ig genes likely to belong to the extrafollicular response (69) while in other diseases, such as rheumatoid arthritis they have been described as memory B cells (70). This novel population of B cells has been found within the memory pool, contributing to inflammation associated with ageing, (‘inflammaging’) through the production of tumour necrosis factor-alpha (TNF-α) (60, 66). ABCs can be stimulated *via* BCR triggering or toll-like receptor (TLR) ligation to secrete pro-inflammatory cytokines (71, 72). Activation of ABCs also induces their differentiation into antibody secreting cells which may contribute to autoimmunity (72). Rubstov et al., showed that CD24- CD38- B cells are present at the onset of autoimmunity and that autoimmune mice depleted of CD24- CD38- B cells, had reduced number of autoantibodies, suggesting that this population plays a major role in the progression of autoimmunity (67, 73). A related population of B cells expressing the IgA receptor FcRL4 in the inflamed synovial tissue expresses RANKL and TNF in the inflamed synovium of patients with rheumatoid arthritis (50, 53, 74).

## B Cells in Liver Disease

The liver is the largest internal organ with a remarkable ability to regenerate upon acute liver damage (75). Dual blood flow to the liver is supplied by the hepatic artery and portal vein, the latter accounting for over 80% of the liver’s blood supply that has passed through the spleen and gut (76). The liver is constantly exposed to gut-derived bacterial products, environmental toxins and food antigens and needs to maintain tolerance in order to prevent an over-active immune response resulting in hepatocyte damage (77–80). Frequent exposure to gut-derived toxins and antigens requires the liver to possess strong innate immune defences despite its constant state of immune tolerance (80–83). However, the liver can shift to a responsive state if an immune response is required (75, 77, 82, 84).

**Figure 2:**
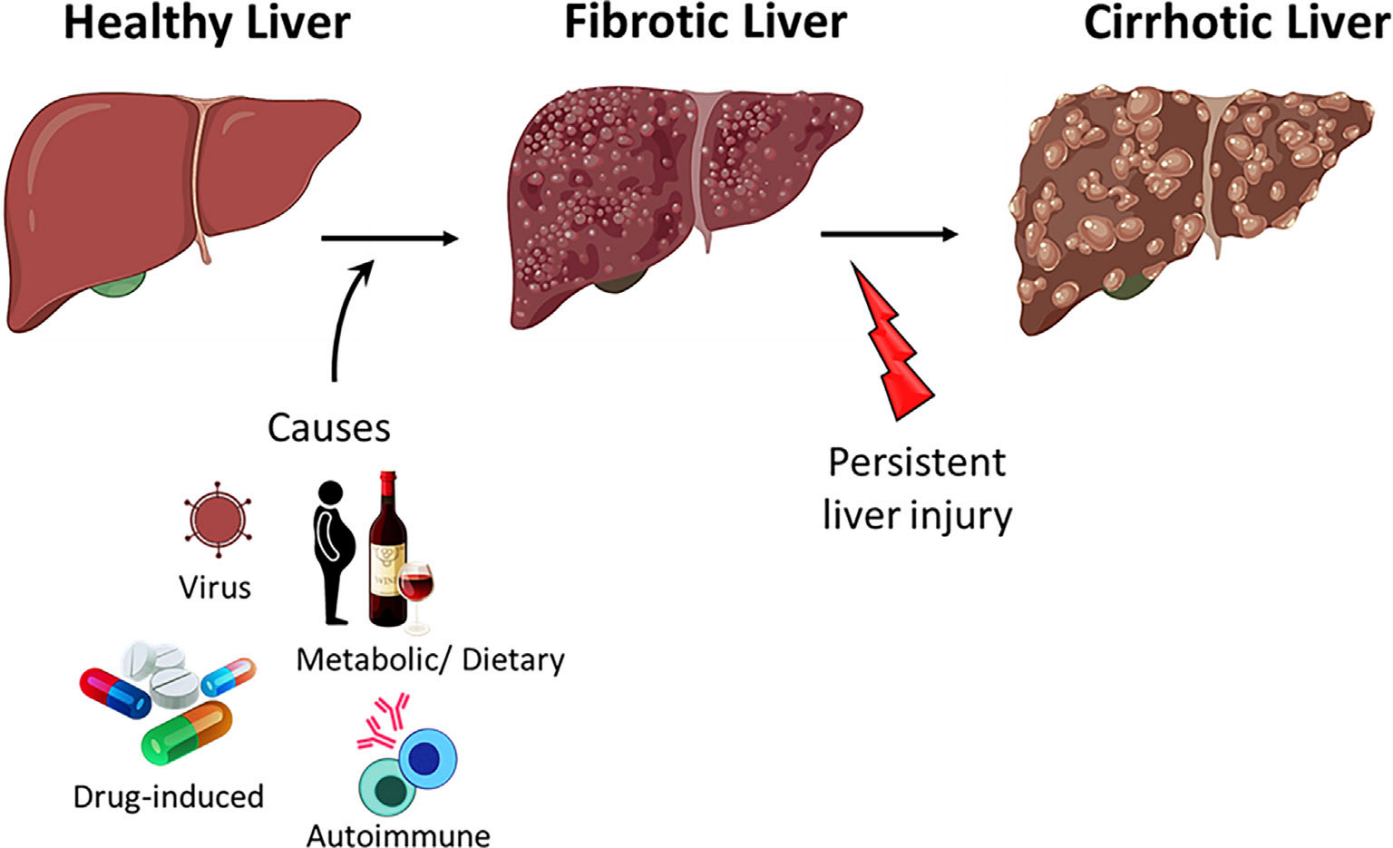
Progression of liver disease. Healthy liver can regenerate after acute injury however, persistent injury to the liver results in hepatocyte damage, inflammation and fibrosis. Persistent insult to the fibrotic liver may progress to cirrhosis.

Acute hepatitis (liver inflammation) resolves upon the clearance of the pathogen or upon elimination of the cause of injury. Failure to clear the infection and resolve the inflammation results in the dysregulation of liver homeostasis and the progression to fibrosis (**Figure 2**) (76). Persistent liver insult can cause chronic inflammation and damage to hepatocytes, which can lead to cirrhosis, the major cause of mortality in chronic liver diseases (CLD) (85, 86). Patients with CLD are also at a higher risk of developing liver cancer (87).

B cells represent up to 50% of intrahepatic lymphocytes in mice with a higher expression of CD5 (88, 89). Novobrantseva et al., showed a role for B cells in fibrosis using carbon tetrachloride (CCL_4_) injections in mice deficient of B cells. B cell deficient mice showed a reduction in fibrotic deposition after 6 weeks of CCL_4_ injections when compared to wild-type mice, *via* accumulation of macrophages that contribute to fibroblast stimulation (88). B cells can contribute to collagen deposition by inducing the differentiation of hepatic stellate cells into myofibroblasts, *via* the production of IL-6 (90). In humans, B cells only account for 8% of the intrahepatic lymphocyte population (76).

Cirrhotic patients will eventually require a liver transplant. Orthotopic liver transplantation (OLT) requires chronic immunosuppressive therapy that can cause post-transplant lymphoproliferative disorders (PTLD) (91). Epstein-Barr virus (EBV) is associated with 60-70% of B cell PLD cases in patients on immunosuppressives (92). The suppressed immune system can no longer control the proliferation of EBV-transformed B cells (92).

### Liver Inflammation in Children

Paediatric immune composition differs to that of adults (93). Dendritic cell and regulatory T cell numbers and functions are decreased in neonates (94). Neonates also have enhanced pro-inflammatory Th17 T cell responses and differences in the Th1/Th2 ratios, all of which could contribute to liver disease (94). The B cell compartment has not been widely characterised in neonatal livers. Duchamp et al., showed significant changes in B cell composition from birth to five years of age in peripheral blood (95). CD27+ IgM+ IgD+ memory B cells formed the largest compartment of B cells in the periphery of paediatric samples (95). B cell populations in paediatric livers remain to be thoroughly examined.

Neonates have incomplete development of the intrahepatic biliary tree and narrow bile ducts which affect the flow of bile and the production of mature bile acids (96). Infants may also have immature hepatocytes that are unable to detoxify and protect the liver from harmful substances (97). Stellate cells were shown to be increased in neonatal rats and they underwent myofibroblastic activation quicker than adult rat stellate cells (98). These may contribute to the rapid progression of liver disease in infants. Examining the B cell compartment in neonates may elucidate immune mechanisms that may contribute to liver disease progression.

We know that some liver diseases are specific to neonates (biliary atresia (97) and others that affect both neonates and adults (non-alcoholic fatty liver disease (NAFLD), autoimmune hepatitis (AIH), primary sclerosing cholangitis (PSC)) however, the differences between paediatric and adult hepatic immunity are poorly understood and not widely studied (96). Few studies have looked at differences in paediatric and adult NAFLD. These studies have found that neonatal NAFLD progressed more rapidly compared to adult NAFLD (99). Furthermore, paediatric NAFLD can be categorised into 2 phenotypes; adult-type (type 1 non-alcoholic steatohepatitis (NASH)) and paediatric-type (type 2 NASH) depending on histology (99, 100). Portal inflammation is mainly seen in children with NAFLD compared to lobular inflammation seen in adults (100). Adults have pericellular fibrosis whereas paediatric NAFLD show portal-periportal fibrosis (100). These discrepancies in histological features may contribute to the rapidly progressing NAFLD in children.

AIH in children presents with a more aggressive course compared to adults. Higher prevalence in females occurs in both paediatric and adult AIH (101). Infants and young children tend to present with type 2 AIH with IgA deficiency and raised levels of IgG (102). Those children with type 2 AIH that are positive for anti-liver kidney microsome type 1 (LKM1) have elevated bilirubin levels and can develop acute hepatic failure within 2-8 weeks of disease onset (102).

### B Cells in Alcohol Related Liver Disease

Alcohol related liver disease (ArLD) is associated with excessive consumption of alcohol causing hepatocyte damage and major shifts in metabolism leading to the retention of fat known as steatosis (86, 103, 104). Cessation of alcohol consumption at the point of early fibrosis and steatosis can reverse ArLD (105, 106). However, continued alcohol abuse can lead to the development of alcoholic steatohepatitis which progresses ultimately to cirrhosis (105–107). The toxic effects of acetaldehyde (the breakdown product of alcohol) cause enhanced lipogenesis resulting in the accumulation of fat molecules in the liver. Continued liver inflammation results in hepatic fibrosis and the formation of scar tissue which disrupts cellular formation (104).

ArLD patients have an altered B cell compartment; significant reductions in immature, memory and naïve B cells were reported in these patients, whilst the percentage of PBs were elevated (103). This increase in PBs may be responsible for high levels of IgA, IgG and IgM in ArLD. It can be hypothesised that a decline in regulatory B cells promotes the release of pro-inflammatory cytokines contributing to the exacerbation of inflammation, further activating immune cells and reducing the inhibitory function of regulatory B cell types (103, 108).

Programmed cell death ligand 1 (PD-L1), constitutively expressed on activated B cells, is the ligand for programmed cell death receptor 1 (PD-1) (109) and interaction between PD-1 and PD-L1 modulate immune responses (110). Kasztelan-Szczerbinska et al., showed a prevalence of PD-1/PD-L1 positive B cells in ALD females when compared to female controls. CD19+ PD-L1+ cells from female ALD patients correlated significantly with all conventional markers of inflammation (109). Sex hormones have been described to influence immune responses. There is evidence that oestrogen can regulate the immune response by modulating B cell function and impairing negative selection of high affinity auto-reactive B cells (111). Females with ArLD also present with elevated titres of circulating immunoglobulins and a variety of autoreactive antibodies (109). Steatohepatitis patients with more advanced disease have reduced numbers of sIgM+, soluble IgG+ (sIgG+) and soluble IgA+ (sIgA+)-reduced memory B cell numbers and increased sIgA+ class-switched memory B cells when compared to healthy controls (103, 108). In addition, alcoholic patients that show no sign of liver disease have a significant expansion of peripheral blood PBs and elevated sIgA+ memory cells (103).

Exposure to alcohol induces immune dysfunction and studies in human and animal models of ArLD show a decrease in B cell numbers (103, 107, 108). An impairment of B cell egress from the spleen to the blood, may account for the reduction in peripheral B cells (103). Despite this decline in B cells, ArLD is defined as an IgA-driven disorder with an increase in IgA complexes, and peripheral blood mononuclear cells (PBMCs) isolated from cirrhotic patients secrete significantly higher levels of IgA that correlate with serum IgA levels (103, 108). Deposition of IgA was observed in different organs and tissues in ArLD patients (103). Factors required for IgA class-switching, such as TGF-β were elevated in chronic ArLD patients together with a T-cell response from T-helper type 2 (Th2) cells (103).

A variety of toll-like receptors (TLRs) are expressed by B cells. TLR ligation activates B cells and is also required for B cell survival, antigen presentation and the production of cytokines and antibodies (112). In alcoholic cirrhosis, TLR-9 activated B cells were associated with a rise in IgA (80). However, Massonnet et al., noted a significant decrease in TLR-9 mRNA expression level in PBMCs from AC patients compared to healthy controls (108). Response to TLR stimulation was diminished in B cells isolated from alcoholic cirrhotic patients whereas, B cells from healthy controls produced IgA upon stimulation with CpG (103). However, B cells isolated from alcoholic cirrhotic patients exhibit an increase in IgA production when stimulated with CpG or R848, a TLR-7 agonist, compared to healthy controls (103). CpG-stimulated B cells, from cirrhotic ArLD patients, secreted more IgA, which may be due to the direct stimulation of B cells (108). B cells from ArLD patients, secreted a mean of 45 times more IgA in the absence of any stimulation compared to B cells from healthy controls (103). These studies show that TLR activation drives liver B cell responses in ArLD.

Alcohol has the ability to downregulate the expression of tight junction proteins permitting the transposition of bacterial constituents and causing a dysbiosis of gut flora, which may contribute to enhanced inflammation due to the presence of higher quantities of dangerous endotoxins (104). Altered intestinal permeability and bacterial translocation is often seen in ArLD patients (103, 107, 108). Impaired intestinal permeability results in the circulation of lipopolysaccharide (LPS), which was increased in the blood of ArLD patients (105, 106). LPS can activate immune cells *via* TLR-4 ligation resulting in further inflammation and damage to hepatocytes in ArLD (86, 105). Furthermore, alcoholic patients have elevated circulating levels of lipopolysaccharide binding protein (LBP) (103, 108). LBP elicits an immune response upon binding LPS, contributing to the inflammatory milieu and hepatocyte damage (105). LPS may trigger the migration of peripheral B cells towards gut-associated lymphoid tissue (GALT). Almeida et al., suggested that chronic alcoholic patients had increased numbers of GALT-derived sIgA+ B cells. This was supported by a significantly higher predominance of IgA+ memory B cells and IgA+ PBs in the peripheral blood of patients (103). In addition, they showed that peripheral blood sIgA+ memory B cells have GC-independent responses, similar to gut lamina propria IgA-producing cells, suggesting that this B cell population is the peripheral counterpart of gut lamina propria IgA-producing B cells (103). These results indicate that LPS, derived from the gut due to alcohol-induced intestinal permeability, could activate immune cells and initiate an inflammatory cascade, further exacerbating inflammation in ArLD.

Increased bacterial translocation results in chronic inflammation which coupled with alcohol abuse, damages hepatocytes (86, 107). Almedia et al., showed a reduction in circulating B cell numbers in ArLD patients; this may be due to alcohol-induced apoptosis of B cells (107). Hepatocyte and leukocyte damage was also mediated by reactive oxygen species (ROS) and acetaldehyde production (a product from the breakdown of alcohol), which destroys cell membranes (86). Bcl-2; a protein that regulates apoptosis, was strongly expressed on B cells in ArLD patients, correlating with the degree of portal and lobular inflammation (107). Significant volumes of cellular debris were produced due to Bcl-2-mediated B cell apoptosis and ROS-induced damage to hepatocytes and biliary epithelial cells (BECs). The release of cellular debris and intracellular proteins from cell debris may activate autoreactive B cells. ArLD patients had autoantibodies against modified liver, suggesting a dysregulated antibody response or impaired negative selection of B cells (105). This may be due to a breakdown in tolerance and a reduction in overall B-reg function.

25-60% of ArLD patients showed the presence of several self-recognising antibodies: mostly antiphospholipid, anti-nuclear, anti-dsDNA and anti-ssDNA (106). These autoantibodies arise due to alcohol-induced oxidative stress which damages cell structures and activates antigen presenting cells (APCs), which recognise haptens; a form of toxic metabolite (106). APCs induce the activation of T cells, which detect both self and non-self proteins, activating B cells to generate antibody secreting cells that release antibodies against proteins and haptens (106). TFH cell numbers were reduced in the blood as a result of excessive alcohol consumption (105). This may be due to the migration of TFH cells to local GC-like structures where they select the survival of B cells, allowing their differentiation into memory B cells and to high affinity antibody producing PCs, which are increased in ArLD patients.

**Figure 3:**
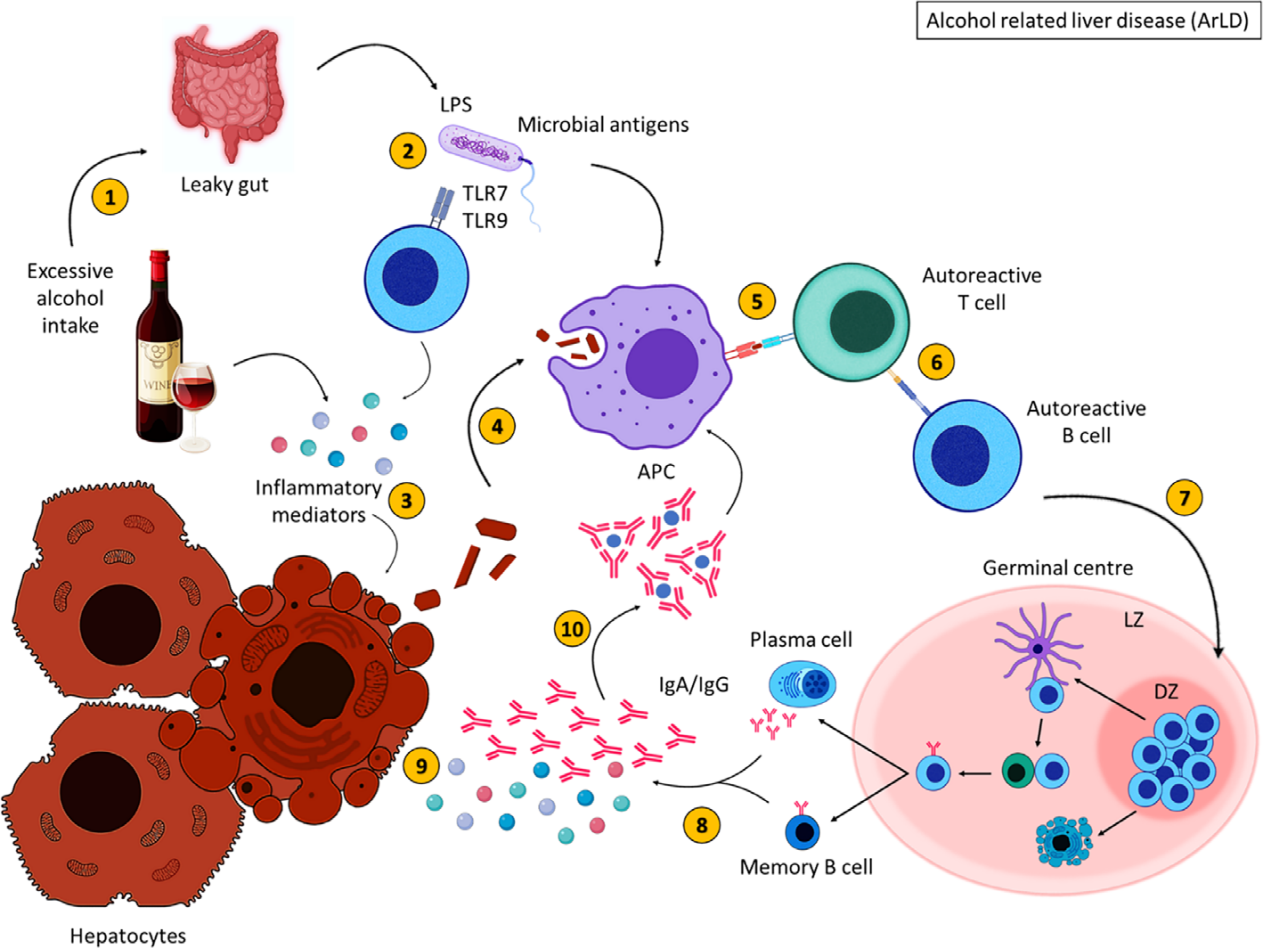
Alcohol related liver disease pathogenesis. Excessive alcohol consumption (1) induces inflammation and results in increased gut permeability (2), allowing bacterial translocation of LPS. Inflammatory mediators damage hepatocytes, resulting in the release of cellular debris (3). Self-antigens are engulfed by antigen presenting cells (4) and presented to autoreactive T cells (5), which stimulate autoreactive B cells (6). Activated B cells then migrate to secondary lymphoid tissues and undergo germinal centre reactions (7) where B cells with increased affinity receptors differentiate into memory B cells and PCs (8). The secretion of inflammatory mediators and autoantibodies from memory B cells and PCs further damage hepatocytes (9). The formation of immune complexes induces further inflammation (10). These immune complexes are engulfed by APCs. Created with BioRender.com.

To summarise, excessive alcohol consumption results in the breakdown of alcohol into acetaldehyde (**Figure 3**). This metabolite induces inflammation and damages cell membranes resulting in the exposure of cellular debris. Alcohol consumption also deregulates the gut barrier allowing bacterial translocation of LPS and other gut-derived pathogens, resulting in the secretion of inflammatory mediators which damage hepatocytes (113). Intracellular antigens from cell debris are engulfed by APCs and are presented to autoreactive T cells that become activated upon antigen recognition; B cells are activated as a consequence of T cell activation, migrate to the GC where they proliferate and differentiate into class-switched memory B cells and antibody secreting cells, with the aid of TFH cells. Increased immunoglobulin secretion ensues, forming immune complexes and further activating the immune response leading to liver injury.

### Non-Alcoholic Fatty Liver Disease

Fat accumulation in the liver causes a range of conditions described as non-alcoholic fatty liver disease (NAFLD) (114). NAFLD can progress from the abnormal retention of lipids in the liver (steatosis) to non-alcoholic steatohepatitis (NASH), where lipid retention is accompanied with hepatic inflammation (114, 115). NASH patients have varying degrees of fibrosis, initiated due to the inflammatory damage of hepatocytes inducing their apoptosis (86). Fibrosis develops to cirrhosis with the eventual requirement of a liver transplant (114, 115). NAFLD patients frequently present with extrahepatic conditions such as obesity, type 2 diabetes, cardiovascular diseases and osteoporosis (115, 116). NAFLD/NASH patients have persistent injury to the hepatocytes due to ROS, lipotoxicity and the secretion of inflammatory mediators from immune cells (115).

The pathogenesis of NAFLD is considered to be a ‘two-hit’ theory; first-hit is the excessive lipid influx and/or a reduction in lipid clearance due to abnormal liver lipid metabolism and the second-hit is the inflammatory process (117), which leads to lobular and portal inflammation and infiltration of activated immune cells (115). Patients with NAFLD had altered hepatic lymphocyte compartments (114), and increased B cells (117) that were associated with disease severity (118). Ectopic lymphoid structures with B cell and T cell aggregates are seen in ~60% of patients with NASH, these aggregates correlate in size and prevalence with lobular inflammation (116). B cells may be involved in fibrosis through the production of inflammatory mediators that stimulate hepatic stellate cells, these cells support liver B cell survival and maturation into plasma cells (116). Isolated B cells from the visceral adipose tissue (VAT) of obese mice show elevated production of pro-inflammatory cytokines whilst a lack of B cells improves fat-induced inflammation (116), suggesting that B cells play an important role in the progression of NAFLD to NASH.

Obese people have altered distribution of adipose tissue. Obesity promotes B cell activation, an early event in the development of experimental NASH animal models, contributing to the progression of steatohepatitis (115). In mice, mesenteric adipose tissue (MAT), located between the gut and liver, affects the liver by secreting inflammatory cytokines, adipocytokines and releasing free fatty acids (FFA) that reach the liver *via* the portal vein (119). B cells from high fat diet (HFD)-fed mice produce IgG and promote epididymal adipose tissue (EAT) inflammation (119). The release of cytokines from inflamed adipose tissue combined with ROS production from dysregulated hepatocyte lipid metabolism, contribute to the progression from steatosis to NASH (86). Intestinal permeability was compromised in NAFLD allowing bacterial translocation and inducing the activation of hepatic inflammatory cells. Patients with NAFLD had elevated serum levels of endotoxin compared to healthy controls (118). Bacterial translocation and LPS promote hepatic inflammation, lipid accumulation and hepatocyte damage (86, 118). Furthermore, hepatic B cells encourage local inflammatory responses when stimulated with LPS (80).

ROS and hepatocyte apoptosis result in the expulsion of hepatocyte cellular debris, inducing antibody production from B cells as a consequence. NAFLD/NASH patients had raised titres of IgG against oxidative stress-derived epitopes (OSE). Patients with increased anti-OSE IgG had a higher prevalence of fibrosis and/or cirrhosis with elevated serum levels of interferon gamma (IFN-γ) (115). PBs upregulate MHC class II as a result of B cell activation in NASH, suggesting that they have a role in presenting OSE to T cells that become activated and contribute to NASH progression (115). Aggregates of B and T cells were observed in 63% of NASH liver samples correlating with the severity of lobular infiltration and enhancement of fibrosis (115). These aggregates were also linked to an increase in anti-OSE IgG titres (115).

**Figure 4:**
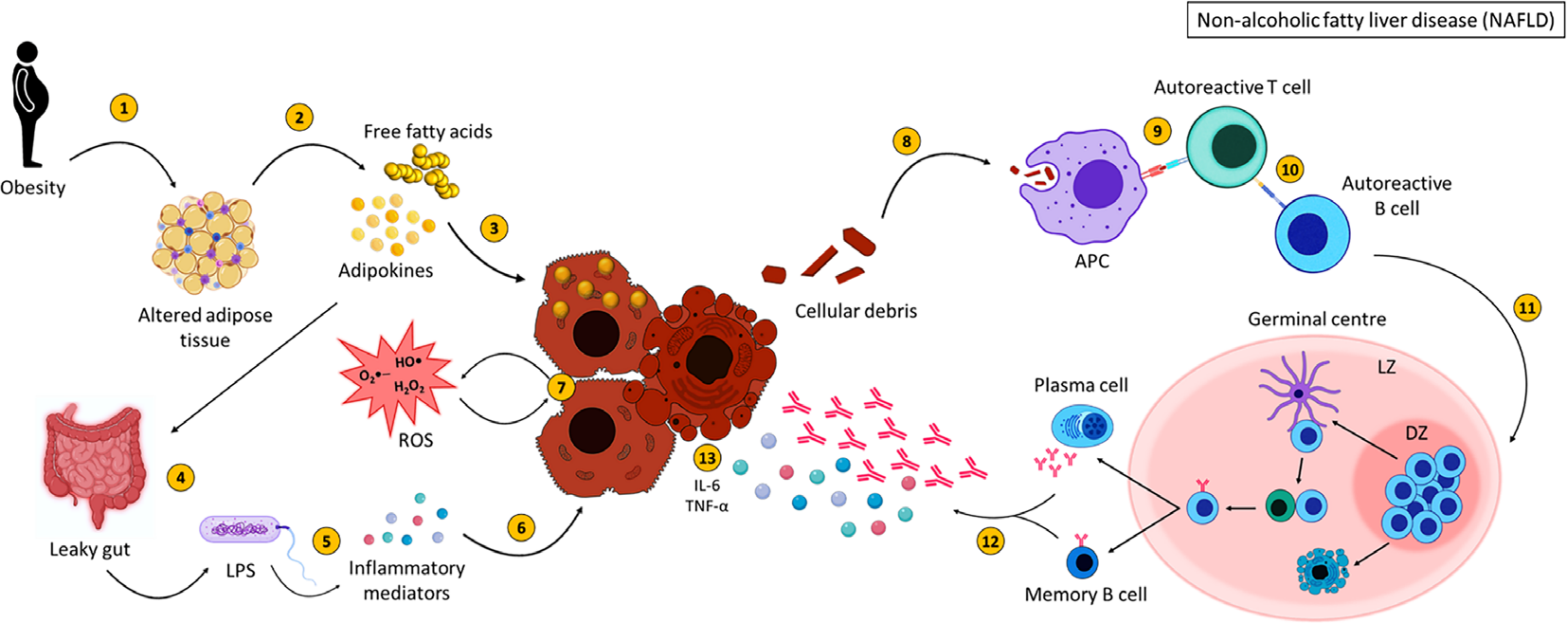
Non-alcoholic fatty liver disease pathogenesis. NASH pathogenesis is linked to obesity and altered adipose tissue distribution (1). Adipose tissue releases adipokines and free fatty acids (FFA) (2), which result in lipid accumulation within the liver (3) and affect intestinal permeability (4). This allows bacterial translocation of LPS and other gut-derived pathogens (5) resulting in the secretion of inflammatory mediators which could damage hepatocytes (6). FFA, adipokines, ROS and inflammatory mediators injure hepatocytes (7) resulting in the expulsion of cellular debris. Self-antigens are engulfed by antigen presenting cells (8) and presented to autoreactive T cells (9), which stimulate autoreactive B cells (10). Activated B cells then migrate to secondary lymphoid tissues and undergo germinal centre reactions (11), where B cells with increased affinity receptors differentiate into memory B cells and PCs (12). The secretion of pro-inflammatory mediators (interleukin-6 and tumour necrosis factor alpha) (120) and autoantibodies from memory B cells and PCs further damage hepatocytes (13). Created with BioRender.com.

To summarise, NASH arises as a result of lipid accumulation within the liver which results in inflammation and fibrosis (**Figure 4**). Activation of various immune cells and the secretion of inflammatory mediators damages hepatocytes, further activating immune cells and initiating an inflammatory loop. B cells produce antibodies against OSE, contributing to increased cytokine production, activation of T cells and the production of ROS, all of which participate in damaging the liver. This vicious cycle of liver destruction results in fibrosis, progressing to cirrhosis making the liver unable to regenerate and heal.

### Viral Hepatitis

There are five types of viral hepatitis, of which hepatitis B and hepatitis C viruses can cause chronic liver disease. Infection may lead to progressive inflammation and liver damage over decades that could lead to end-stage disease requiring a transplant, however these viruses are not directly cytopathic (75, 121).

Antibodies against both viruses are generated in infected individuals. In HBV, IgM antibodies against the core protein are used as a marker of early infection whereas, antibodies against the hepatitis surface (HBsAg) and e (HBeAg) antigens appear as the infection progresses (121, 122). HBsAg, a T cell-independent antigen, induces the activation of naïve B cells and mounts a robust antibody response (71, 123). Adults infected with HBV are able to resolve infection in the majority of cases, and there is an effective vaccine for HBV based on the HBsAg (124). Chronic HBV patients have circulating HBsAg-specific B cells, however these cells are unable to produce effective anti-HBs antibodies (71). The early humoral immune response in HCV is difficult to study as many patients are asymptomatic (125).

Intriguingly, antibodies to the envelope glycoproteins in HCV infection may emerge late and are abundant in chronic patients, while at the same time showing potent neutralising ability of heterologous viruses. Investigations in autologous virus neutralisation over years in a single patient showed that this RNA virus remained a step ahead of the antibody response by generating variants to escape neutralising antibodies (126). However, antibodies can prevent HCV infection *in vivo* and contribute to the eradication of the HCV infection (127). There is no vaccine against HCV infection, but immunisation of healthy volunteers with viral envelope glycoproteins resulted in the generation of neutralising antibodies (128), and antibodies were shown to be protective in a human liver chimeric mouse model (129). Immunisation of genetically humanised mice with soluble envelope glycoprotein 2 (sE2), derived from insect cells, produced high titres of broadly neutralising antibodies against diverse HCV envelopes and were protected from HCV infection, *in vivo*. Immunisation of non-primates with insect derived sE2 resulted in the induction of B and T cell immunity (127). The role of B cells and antibodies in the context of failure to control HCV infection was elegantly described by Dustin et al., (130).

An accumulation of circulating B cells within the liver is associated with severe liver damage (131) and elevated levels of activated B cells is seen in patients with HBV and HCV (121). However, these cells have a reduced proliferative capacity and express Fc receptor-like protein 4 (FcRL4), an inhibitory receptor overexpressed on exhausted memory B cells (121). This suggests that B cells are dysfunctional in infected livers as they are chronically activated and adopt an exhausted phenotype. Hepatic release of subviral particles (empty virions consisting of mostly HBsAg) (132) is an immune evasion mechanism in HBV which forms immune complexes by crosslinking neutralising antibodies targeting the virus. This leads to continual BCR triggering, promoting the expansion of exhausted memory B cells, also referred to as atypical memory B cells (71, 123, 133). Chronic hepatitis B patients had deposits of HBcAg-immune complexes in their liver (134). Fc receptor-like protein 5 (FcRL5) suppresses the activation of B cells by crosslinking to immune complexes and PD-1 inhibits B cell signalling; both these markers were enriched on the surface of atypical memory B cells; T-bet is also associated with the generation of atypical memory B cells (71, 135). This population of atypical memory B cells was found to be present in infected livers (71) Atypical memory B cells enriched in HBV were unable to escape apoptosis and differentiate into effective HBsAg-specific antibody secreting cells (71), impairing their ability to produce neutralising antibodies against the viruses (123, 133, 134).

IL-10 producing B-reg cells are another subset of regulatory B cells that are enriched in HBV and HCV patients, which may contribute to viral persistence (71, 121, 136–138). Eiza et al., showed an increased in IL-10 producing B-regs in chronic HBV patients, when compared to healthy controls and these cells were able to dampen down HBV-specific CD8+ T cell responses (138). A subset of B-reg cells that express high levels of CD5, CD1d and IgD are thought to be responsible for IL-10 production by B cells (136, 138). CD5+ B-regs produce IL-10 upon activation and correlate with poor virus elimination (138).

Beyond immune surveillance, we previously showed that B cells were vehicles for HCV transmission to hepatocytes (139). Stimulated B cells were able to bind viral particles using scavenger receptor B type 1 and C-type lectins DC-SIGN and L-SIGN and internalised the virus in compartments that prevented virus degradation. The intact virus was then recycled to the B cell surface within hours. B cell-transmitted virus was more infectious than cell-free virus, adding a pathogenic role for B cells in HCV infection. HCV RNA was detected in 83% (110/132) of patients with HCV genotype 1 (140). Inokuchi et al., reported that HCV RNA was detected more frequently in B cells compared to CD4+ and CD8+ T cells (141). The role of antibodies and adaptive immunity in HCV infection has been recently reviewed (142–144).

The most common B cell lymphoproliferative disease associated with HCV is mixed cryoglobulinemia (MC) (145, 146). MC presents with formations of cryoglobulins; abnormally precipitated immunoglobulins that can be coupled with rheumatoid factor (147), detected in the circulation of 40-60% of HCV-infected patients (148, 149). B cells contribute to the formation of cryoglobulins through uncontrolled autoantibody production and proliferation (149). These cryoglobulin-containing immune complexes deposit in small or medium vessels causing vasculitis (145, 150, 151). Whilst cryoglobulinemia is common in HCV, rare cases have been reported to exist in HBV infected patients (151). The clonal proliferation of B cells in MC (152), may cause the formation of ectopic lymphoid aggregates within the liver of HCV patients. Lauletta et al., has shown that cytokine (CXCL13) can cause B cell migration to intraportal lymphoid aggregates in the liver and create a microenvironment to sustain B cell aggregation (153).

### Autoimmune Hepatitis

Autoimmune hepatitis (AIH) is a chronic autoimmune disorder requiring life-long immunosuppressive therapy (75, 154–156). This disease affects all ages, races and sexes although it has a higher prevalence in females (75, 157–159). AIH is associated with other autoimmune diseases such as coeliac disease and can coexist with autoimmune family biliary liver diseases; primary biliary cholangitis (PBC) or primary sclerosing cholangitis (PSC) (159). This progressive, necro-inflammatory disease is linked to increased immune infiltration that destroys the hepatic parenchyma through immune-mediated hepatocyte damage (75, 107, 154, 155, 157, 158, 160, 161). Fibrosis and cirrhosis are ramifications of chronic inflammation and 40% of AIH patients present with cirrhosis at the time of diagnosis (154). Despite the use of corticosteroids and immunosuppressives, 10-20% of patients with AIH will progress to end-stage liver disease requiring liver transplantation (160).

AIH classification is dependent on antibody specificity. Patients with AIH can have numerous autoantibodies (162), including antinuclear antibodies (ANAs), smooth muscle antibodies (SMA) and antibodies directed against liver kidney microsome type 1 (LKM1) (107, 157, 159, 163). Type 1 AIH is characterised by the presence of ANA, SMA and perinuclear anti-neutrophil cytoplasmic antibodies (pANCA), the latter is present in 65-92% type 1 AIH patients (157, 159). Autoantibodies against liver cytosol type 1 (LC1) and/or anti-LKM1 antibodies are classified as type 2 AIH (159, 160); pANCA antibodies are not present in this type of AIH. CYP2D6 is the antigen for anti-LKM antibodies and anti-LC1 antibodies target a liver-specific metabolic enzyme, formiminotransferase cyclodeaminase (FTCD) (157, 160). CYPD26 autoantibodies are of the IgG isotype, supporting the role that T-dependent class-switching is essential to produce IgG+ PCs (158). Anti-LC1 antibody titres are associated with disease severity and are detected in 30-50% of patients with type 2 AIH (157). Type 3 AIH is proposed to be defined by the presence of anti-soluble liver antigen/liver pancreas antigen antibodies (anti-SLA/LP antibodies) which are present in 10-30% of AIH patients (157). 50-76% of AIH patients have antibodies against the asialoglycoprotein receptor (ASGPR) which is a component of the liver specific lipoprotein (LSP) expressed on hepatocyte surfaces (157, 163). Disease activity and poor outcome of AIH positively correlated with titres of anti-ASGPR in this group of patients (157).

Hepatic destruction in AIH is thought to be driven by T cells, however, the presence of several autoantibodies suggests a role for B cells in the pathogenesis of AIH (154). Elevated serum IgG levels are found in up to 85% of patients with AIH displaying ongoing inflammation within these patients (164). AIH liver biopsies showed mixed infiltration of T cells and B cells, including IgG+ B cells and PCs (157–159, 163, 165). AIH flare ups show an increased number of T and B cells present within the liver (159). B cells present self-antigens to autoreactive T cells which become activated, which then stimulate B cells to produce autoantibodies (163). Increased expression of CD86 is seen on B cells from new onset AIH patients, suggesting that these B cells are primed to co-stimulate T cells (166). Cytokines produced by Th2 cells also aid in B cell activation and differentiation *via* IL-4 production, which was elevated in AIH (157, 163).

IgG+ cells were significantly higher in AIH liver samples and were found distributed around the bile ducts and in portal tract areas, along with IgM+ cells (157, 158, 161, 165). Lymphocytes target IgG bound to hepatocytes in healthy individuals, mediating cellular injury and initiating inflammation (75).

Patients with systemic lupus erythematosus (SLE), an autoimmune disease driven by a dysregulated B cell response, frequently develop inflammation of the liver. Hepatic dysfunction in SLE patients can be caused independently of B cell responses for example by side effects of medication. However, two key examples for autoimmune liver conditions associated with SLE are lupus hepatitis (also known as SLE-associated hepatitis) and autoimmune hepatitis. Both involve extensive B cell activation and are often difficult to distinguish. They are both associated with hyperglobulinaemia but show differences in the profile of autoantibodies. Since their prognosis and therapeutic approach differs, it is an important goal to develop safe diagnostic criteria (167).

AIH is an autoimmune disease with many factors contributing to disease progression, however the trigger is unknown. The presence of autoantibodies, targeting many self-proteins, presents an important role for autoreactive B cells in the pathogenesis of AIH and suggests an impairment in central B cell tolerance. The survival and activation of autoreactive T and autoreactive B cells is a result of a breakdown in self-tolerance and a reduction in immune regulation.

### Primary Sclerosing Cholangitis

Primary sclerosing cholangitis (PSC) is a cholestatic autoimmune disease in which fibrosis and chronic inflammation destroy the large bile ducts (157, 168–170). PSC is associated with inflammatory bowel disease (IBD); 87% of PSC patients present with ulcerative colitis (UC) and 13% have Crohn’s disease (CD) (83, 157, 168). Chronic destruction and scarring of the biliary tree leads to cirrhosis and many patients will eventually require liver transplants (169).

Anti-neutrophil cytoplasmic antibodies (ANCA) are detected in 88% of PSC patients however, these autoantibodies are not specific for PSC, but also seen in AIH and biliary atresia (BA) (157); PSC-specific autoantibodies have not been identified to date, but disease-relevant epitopes have been detected (171). PSC disease severity is associated with concentrations of anti-cardiolipin antibodies which were present in 2/3 of PSC patients (157).

Total numbers of B cells were significantly higher in PSC-derived PBMCs compared with healthy controls (64). Furthermore, 10% of PSC patients had elevated serum levels of IgG4 and a significant infiltration of IgG4 PCs (169, 172). IgG4+ PC aggregates were observed in PSC tissues and IgG4+ deposits were reported (169, 173). Fischer et al., showed that the intensity of IgG4+ immunostaining was linked to disease progression and infiltration of lymphocytes in PSC (169). B cells isolated from PSC liver explants produce a range of autoantibodies when cultured suggesting, that the targets in PSC are self-antigens or arise as a result of cross-reactivity of exogenous targets (168). Approximately 50% of explanted PSC liver specimens displayed evidence of IgG4+ cells and these tissue infiltrating IgG4+ cells were associated with a clinically aggressive disease course and a higher probability of liver transplantation (169). IgG4-related disease (IgG4RD) is an inflammatory disease associated with elevated numbers of IgG4-positive PCs which contribute to chronic damage and fibrosis (174, 175). IgG4RD-associated sclerosing cholangitis can be mistaken for PSC, which may explain the increase in IgG4+ cells seen by Fischer et al. (176).

70% of PSC patients have IBD which is linked to defects in the intestinal barrier (177). The gut microbiota was altered in PSC patients when compared to UC and healthy controls (168). Gut-derived antigens may trigger the autoimmune response in PSC by allowing the translocation of bacterial and food antigens (78, 168). BECs propagate their own destruction when they are stimulated by LPS, which induces them to release chemokines and cytokines (83). These mediators activate various immune cells which damage the tissue leading to fibrosis and resulting in an inflammatory cascade (83). Other gut-derived bacterial motifs also stimulated BECs to drive their own destruction and analysis from PSC livers showed the presence of bacterial RNA (83).

The pathogenesis of PSC is reviewed by Lleo et al. (178). PSC may be initiated by a loss of self-tolerance due to bacterial antigens and the obliteration of BECs, resulting in the expulsion of self-antigens which activates autoreactive immune cells. Molecular mimicry may contribute to this initial loss in tolerance. Primed gut-derived T cells migrate to the liver where they may induce B cell proliferation and differentiation into IgG4+ secreting PCs (83). These immune cells will secrete many pro-inflammatory cytokines contributing to inflammation, the destruction of BECs and the progression of autoimmunity.

### Primary Biliary Cholangitis

Primary biliary cholangitis (PBC) is a progressive autoimmune disease characterised by immune-mediated destruction of the intrahepatic small bile ducts (79, 107, 157, 179–182). This deregulated immune response results in liver inflammation and damage, causing fibrosis and eventually cirrhosis as an outcome of the accumulation of bile toxins (79, 183, 184).

There is a profound loss of B cell tolerance associated with PBC, which is supported by the presence of autoantibodies (83, 180, 185, 186); 90-95% of PBC patients have the presence of specific anti-mitochondrial antibodies (AMA), directed against the mitochondrial inner membrane member, 2-oxoacid dehydrogenase complexes (2-OADC) (79, 107, 157, 179, 181–183, 185–187). Autoantibodies targeting the E2 subunit of the pyruvate dehydrogenase complex (PDC-E2) is a major autoantigen in PBC (157). 50% of PBC patients had antibodies targeting the nuclear pore complex members; gp210 and p62 (157). The anti-nuclear antibodies (ANAs) targeting gp210, correlate with disease severity (79, 157, 186). PBC patients had significantly higher PDC-E2 specific IgM, IgG, and IgA PB frequency (64). In addition, many PBC patients present with hyper-IgM expression in their serum (179, 186). Complement activation *via* agglutination by IgM plays a crucial role in innate immunity providing a link between innate and adaptive immunity as IgM enhances antigen-driven IgG responses (179).

GCs are essential for the production of class-switched immunoglobulins however, they also allow the differentiation of autoreactive B cells into autoreactive memory and autoantibody producing PCs in PBC (183, 185, 186, 188). TFH cells promote GC formation and allow B and T cell interaction promoting B cell activation, proliferation and differentiation into affinity matured, long-lived PCs (79, 183). TFH locate B cell follicles *via* the CXCL5 – CXCL13 chemokine axis and were present in vast numbers near damaged bile ducts, in lymphoid follicle-like structures (79). In healthy control livers, hepatic TFH cells were absent (183). PBC-derived TFH cells had a greater ability to induce B cell differentiation into class-switched memory B cells and mature PCs (183). Circulating TFH (cTFH) cell frequency was higher in PBC patients and in patients who do not respond to ursodeoxycholic acid (UDCA) treatment compared to UDCA responders (79). cTFH cells positively correlated with circulating PCs in PBC and secrete high levels of IL-21 inducing B cell proliferation, differentiation and secretion of autoantibodies suggesting that TFH cells contribute to PBC pathogenesis (79, 183, 185). Increased serum IL-21 levels positively correlated with concentrations of serum AMA and IgM (185). IL-21 is vital for the development of TFH cells and induces maturation of B cells in a paracrine manner whilst enhancing TFH function in an autocrine fashion (79, 183).

Tissue from the livers of PBC patients showed the presence of several bacterial products (83). TLR signalling pathway was activated in PBC patients and hyper IgM production which may be due to increased bacterial infections (83, 157). Studies have shown that the induction of PBC occurs due to molecular mimicry between PDC-E2 and bacterial proteins (79). Molecular mimicry may be the initial insult in the loss of self-tolerance, enabling the survival of autoreactive B cells that fail to enter apoptosis (186). Activation of TLRs induces the proliferation of B cells and the secretion of pro-inflammatory cytokines. TLR-9 expression was increased in B cells from PBC patients and CpG stimulation enhanced the secretion of IgM, cytokines and chemokines (83, 157, 179, 187). Kikuchi et al., showed a positive correlation between the intensity of TLR-9 expression and IgM+ memory B cells (83, 179). Bacterial motifs were required to increase TLR-9 expression on B cells and promote inflammation (83, 179). Furthermore, CpG stimulation of PBMCs derived from PBC patients resulted in vast production of AMAs compared to unstimulated controls (157). TLRs are also expressed by cholangiocytes which aid in immune activation and may contribute to PBC pathogenesis. TLR-4 and TLR-9 levels were highly expressed on cholangiocytes in PBC patients (75). Ma et al., showed increased TLR-4 expression on BECs in PBC and expression was seen in periportal and interlobular hepatocytes in patients with advanced disease (83).

BEC themselves may contribute to the initiation and progression of PBC rather than being the victims of the immune response. Damage to BEC is a hallmark of PBC and BEC obtained from PBC livers rapidly undergo apoptosis (186). BEC can engulf apoptotic BECs and translocate PDC-E2 into apoptotic bodies (186). The immunologically intact PDC-E2 is presented to autoreactive immune cells initiating their activation, secretion of pro-inflammatory mediators and AMA production (186).

Many factors contribute to the initiation and pathogenesis of PBC which is reviewed Carbone et al., (189). The initial insult in PBC is thought to be similar to that of PSC; molecular mimicry by bacterial motifs, subsequently activating the immune response and breaking down self-tolerance. The inflammatory milieu is further exacerbated by the destruction of BEC, which further activate autoreactive immune cells *via* antigen presentation of PDC-E2 in apoptotic bodies. The ongoing inflammatory cascade results in additional destruction of bile ducts, activation of autoreactive immune cells and the production of autoantibodies.

### Biliary Atresia

Biliary atresia (BA) affects 1 in 8,000-18,000 neonates and encompasses a host of potential aetiologies leading to progressive liver damage (190–192). Obliteration of the extrahepatic biliary tree and subsequent progressive destruction of the hepatic ducts leads to fibrosis and cirrhosis in BA infants (192, 193).

There are two forms of BA; acquired and congenital (194, 195). 80% of BA patients have the acquired form and 20% have the congenital form, both are characterised by destruction of bile ducts and fibrosis, with various degrees of inflammation (191, 195, 196). BA infants with the congenital form also present with other genetic abnormalities (195, 196). Kasai portoenterostomy (Kasai) is a surgical treatment performed at diagnosis in over 95% of BA infants (190). The Kasai procedure removes the damaged bile ducts and anastomoses the jejunum to patient intrahepatic bile ducts to allow bile flow from the liver to the gut; despite successful surgery, 80% of BA patients will require a liver transplant (191, 197). Medical management post-Kasai involves the use of antibiotics, vitamin supplementation, nutritional support and administration of UDCA to encourage bile flow (192). Kelly and Davenport show that having specialised centres for portoenterostomy surgery has improved survival to over 90% in the UK. This study also showed a reduced need for liver transplantation due to the centralisation of surgery (192).

BA livers showed increased immune infiltration and elevated lymphocyte activation in the portal tracts (191, 193). There was an increased presence of intrahepatic periductal B cells in BA patients at diagnosis and at the time of transplant (190). These activated B cells secrete IgM and IgG antibodies and Lu et al., found that IgG from the sera of BA patients reacted with the cholangiocyte cytosol (198, 199). Furthermore, 40% of BA infants had deposits of IgM and IgG along the basement membrane of the bile duct epithelia (190, 198, 200). Infants with BA show increased levels of high-affinity pathogenic IgG antibodies and a reduction in the level of natural IgM, which plays a protective role in immune function and the development of autoimmune disease (201). Anti-α-enolase and ANCA autoantibodies are observed in BA neonates and were detected in the sera of BA patients (190, 198). Anti-α-enolase IgM and IgG antibodies can be found in BA children who still have their own livers suggesting a role for B cells in BA pathogenesis (199).

There are various animal models of BA (195, 202) however the commonly used model is the rhesus group A-rotavirus (RRV)-induced mouse model of BA (193, 199, 203, 204). RRV-induced mice are able clear the virus by 2 weeks however, they show signs of extrahepatic bile duct obstruction and progressive inflammation, which leads to liver failure (199). Despite the evidence of viral insult in mouse models of BA, there are conflicting studies detecting the presence of rotavirus in BA patient samples. One study shows the presence of type C rotavirus RNA in 10 out of 20 BA liver samples (205) whereas Bobo et al., did not detect any rotavirus RNA from their BA liver cohort (n=10) (206).

**Figure 5:**
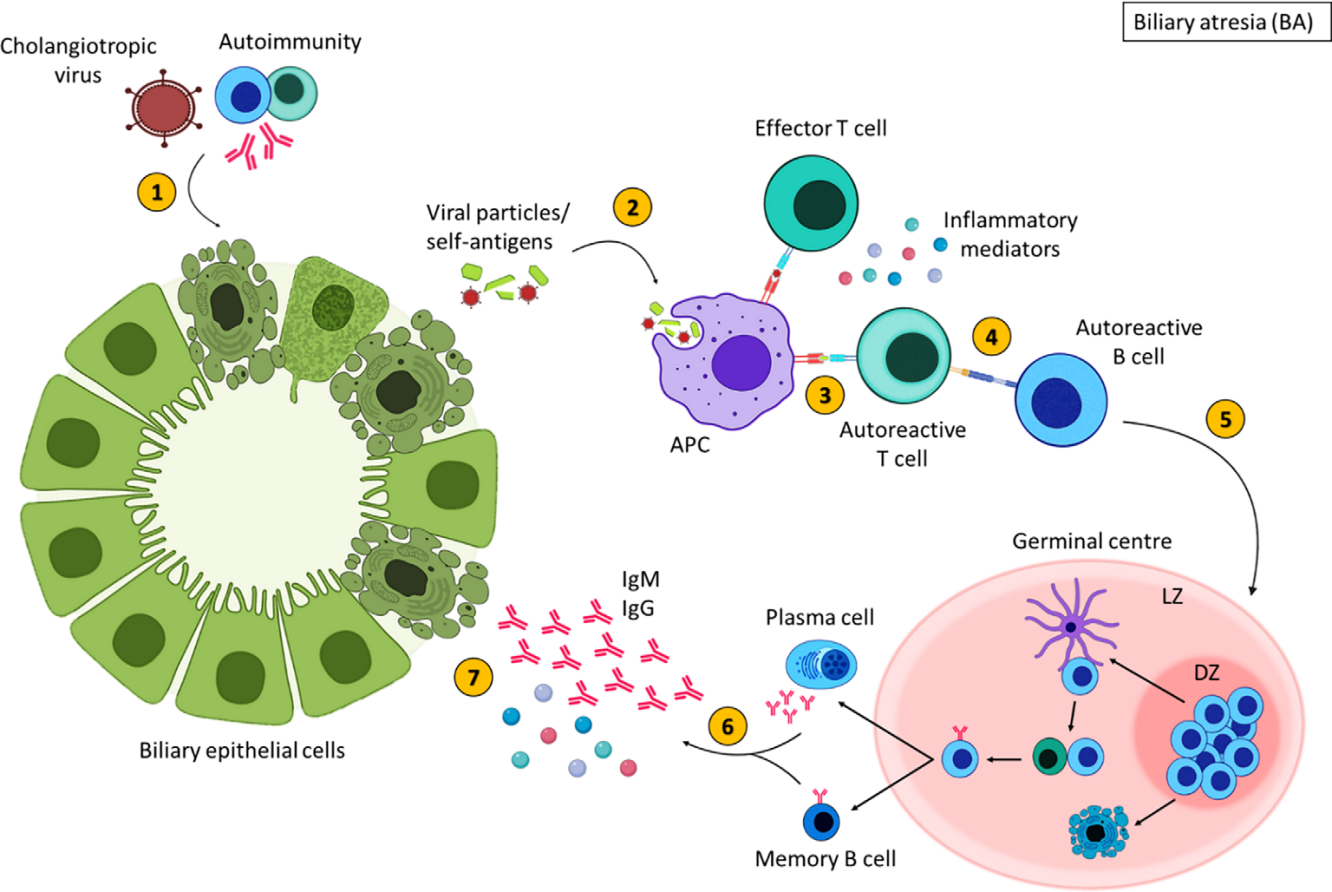
Inflammatory-mediated damage in biliary atresia. In some children with BA, damage to the extrahepatic bile ducts may occur due to cholangiotropic viruses or autoimmunity (1), resulting in the expulsion of viral or self-antigens. These antigens are engulfed by antigen presenting cells (2) and presented to T cells (3). Autoreactive T cells that recognise self-antigens stimulate autoreactive B cells (4). Activated B cells then migrate to secondary lymphoid tissues and undergo germinal centres reactions (5) where B cells with increased affinity receptors differentiate into memory B cells and PCs (6). The secretion of inflammatory mediators and autoantibodies from memory B cells and PCs further damage BECs (7). Created with BioRender.com.

To summarise the trigger for BA is unknown; viral, environmental, genetic and autoimmune factors are thought to contribute to BA pathogenesis (196, 198). A proposed theory for the pathogenesis of some types of BA is an initial infection with a cholangiotropic virus which may damage the bile duct epithelia directly, however this virus is still unidentified (195) (**Figure 5**). This initiates an immune response resulting in an exaggerated inflammatory response that further damages BEC (196). The injured bile ducts release altered self-antigens and may express self-antigens on their surface (194, 207). APCs recognise these self-antigens as foreign molecules subsequently, activating autoreactive T cells, mediating inflammatory destruction of the bile ducts (194). Activated autoreactive T cells also stimulate autoreactive B cells, augmenting the production of inflammatory mediators and initiating B cell differentiation. Despite the clearance of the virus, persistent inflammation contributes to the obliteration of the bile ducts leading to fibrosis and liver failure (199). It is important to stress, that inflammation is evident in a subsection of patients, and some children with BA show no inflammatory histological findings at Kasai or at end stage disease explant tissue. Histological characterisation of the immune compartment in BA may aid our understanding of disease pathogenesis.

**Table 2** provides a brief summary of clinical features and immune involvement in liver diseases.

**Table 2:** Key clinical features and immune involvement in liver diseases.

Disease	Clinical features	Immune involvement	References
Alcohol related liver disease (ArLD)	Hepatocyte damage Steatosis Fibrosis Cirrhosis Lipogenesis Accumulation of fat in the liver High levels of IgA, IgG and IgM Lipopolysaccharide circulation Portal and lobular inflammation	Liver inflammation Altered B cell compartment Increased plasmablasts Decreased regulatory B cells Reduction in circulating B cells	(86, 103–108)
Non-alcoholic fatty liver disease (NAFLD)	Steatosis Hepatic inflammation Fibrosis Hepatocyte damage Cirrhosis Lipid influx Portal and lobular inflammation Altered distribution of adipose tissue Elevated levels of endotoxin Raised IgG titres	Liver inflammation Damage by reactive oxygen species, lipotoxicity and inflammatory mediators Infiltration of activated immune cells Increased B cells associated with disease severity Ectopic B and T cell aggregates LPS stimulates B cells to secrete inflammatory mediators	(80, 86, 114–118)
Viral hepatitis	Antibodies against viral epitopes Formation of immune complexes	Progressive inflammation and liver damage Accumulation of circulating B cells within the liver Elevated levels of activated B cells Dysfunctional B cells Expansion of exhausted memory B cells Enrichment of atypical B cells Increase in IL-10 producing regulatory B cells B cells can act as vehicles for HCV transmission	(71, 75, 121–123, 131, 133, 134, 136, 138, 139)
Autoimmune hepatitis (AIH)	Associated with other autoimmune diseases Necro-inflammatory disease Destruction of the hepatic parenchyma and hepatocytes Fibrosis Cirrhosis	Increased immune infiltration Presence of autoantibodies Elevated serum IgG levels B cells are primed to co-stimulate T cells *via* CD86 interaction	(154, 159, 162, 164, 166)
Primary sclerosing cholangitis (PSC)	Fibrosis Destruction of the large bile ducts Associated with IBD Cirrhosis Destruction of the biliary tree Defects in intestinal barrier Altered gut microbiota	Presence of autoantibodies High numbers of B cells IgG4+ plasma cell aggregates and deposits in some PSC patients	(64, 157, 168–170, 172, 177)
Primary biliary cholangitis (PBC)	Affects small bile ducts Fibrosis Accumulation of bile toxins Presence of several bacterial products	Immune-mediated destruction of intrahepatic small bile ducts Liver inflammation Loss of B cell tolerance Presence of autoantibodies Hyper-IgM expression in the serum Complement activation *via* agglutination by IgM	(83, 157, 179, 181, 184, 186)
Biliary atresia	Progressive liver damage Obliteration of the extrahepatic biliary tree and hepatic ducts Fibrosis Cirrhosis	Increased immune infiltration Elevated lymphocyte activation in the portal tracts Increased presence of intrahepatic periductal B cells IgM and IgG deposits High levels of high-affinity pathogenic IgG antibodies Autoantibodies may be present	(190, 191, 193, 199, 201, 207)

A brief summary of the clinical features and immune compartment involvement in adult and paediatric liver diseases.

### Targeting B Cells in the Liver - Rituximab Treatment

Originally developed for the treatment of B cell lymphoma, rituximab is a human/murine chimeric monoclonal antibody that targets specifically the cell surface glycoprotein CD20 (208). CD20 is universally expressed by normal B cells through all stages of development from late pre-B cells in the bone marrow and right before terminal differentiation to plasma cells.

The true role of CD20 remains poorly understood; it has no known natural ligand, however its association with the BCR suggests a role in B cell signalling. CD20 is not immediately internalised upon antibody binding (209, 210), and thus monoclonal antibodies raised against it cannot be used to deliver cytotoxic moieties into the cell. As a result, the mode of action of anti-CD20 antibodies relies on the subsequent recruitment of the host immune response to opsonisation.

Multiple modes of actions have been proposed for rituximab mediated B cell depletion. Rituximab colocalises CD20 to lipid rafts (211), and through this induces B cell killing by NK cells through antibody-dependent cellular cytotoxicity (212). Efficacy of rituximab, however, differs greatly among different autoimmune diseases. Amongst these, Rituximab is approved for treatment of rheumatoid arthritis, granulomatosis with polyangiitis and microscopic polyangiitis and pemphigus vulgaris (213–215). In SLE, pilot trials and observational studies were initially promising but larger scale clinical trials did not show a clear benefit. Recent trials of a combination of Belimumab, which targets the cytokine BLyS with Rituximab, however, show promise in SLE (216). Direct cross-linking of CD20 on B cell tumour cell lines was shown to be sufficient for the induction of apoptosis through MAP kinase activation (212, 217). Rituximab may also induce complement dependent cytotoxicity (212, 217, 218). In a mouse model, Kupffer cells within the hepatic sinusoids have been shown to capture anti-CD20 antibody coated B cells (219).

During differentiation into mature antibody-secreting plasma cells, CD20 expression is lost (220). Due to this absence of CD20, rituximab treatment does not affect the production of long-lived PCs (221, 222), as rituximab does not deplete long-lived PCs. In multiple diseases where rituximab treatment has been trialled, 90-100% of peripheral B cells were depleted (223).

Efficacy of rituximab, however, differs greatly among different autoimmune diseases. Amongst others, rituximab has been shown to be an effective treatment for rheumatoid arthritis (224), systemic lupus erythematosus (225), thrombocytopenic purpura (226), and autoimmune haemolytic anaemia (227). B cells have now begun to be targeted in CLD (3).

### Rituximab in Viral Hepatitis

Rituximab has been shown to be the most widely used treatment for HCV patients with cryoglobulinemia vasculitis (148, 228). One cycle of low-dose rituximab achieved a complete clinical response in 22 out of 31 (70.96%) of MC patients (229). Clinical manifestations of cryoglobulinemia such as skin ulcers, renal manifestations and sensitive-motor neuropathy have improved through the use of rituximab (228). Rituximab treatment reduces serum levels of cryoglobulins and rheumatoid factor through the clonal B cell depletion in the bone marrow (230).

### Rituximab in Autoimmune Hepatitis

Non-specific immunosuppression using prednisolone and azathioprine has improved symptoms and subsequently survival in patients with AIH (231). However, some patients either develop adverse side effects and as a consequence discontinue treatment or exhibit a suboptimal response to this standard therapy (232). As a result, more targeted immunotherapies for this disease are needed.

In a mouse model of AIH, administration of anti-CD20 antibodies resulted in a significant reduction in liver inflammation and ALT levels, but there was no reduction in the total IgG levels or autoantibody titres (233). The depletion of B cells resulted in a significant increase in naïve CD4^+^ and CD8^+^ T cells and a reduction in antigen-experienced T cells. In this model of AIH, B cells played an active role in disease pathogenesis through the antigen presentation process and modulated T cell functions (233).

Rituximab has been trialed in both adult and paediatric patients with AIH which was unresponsive to prior treatments (234, 235). Rituximab was well tolerated, and complete remission was achieved and maintained. Serum IgG levels were also reduced, and ANA titres were decreased in 2 out of 6 subjects, becoming negative in one (234). More recently a multicentre retrospective study reported clinically meaningful reductions in liver enzyme values following the administration of rituximab in 22 patients with difficult to manage AIH (236). After treatment, 71% of patients were free from AIH flares (236).

### Rituximab in Primary Biliary Cholangitis

Currently, therapy for PBC is limited to UDCA and, for patients with end-stage liver disease, liver transplantation. Although UDCA has demonstrated clinical benefits in liver biochemistries (237), up to 40% of patients have a suboptimal response to UDCA and 10% will go on to die or require liver transplantation (238).

At present, trials for the efficacy of rituximab in PBC have primarily enrolled patients who have demonstrated an unsatisfactory response to UDCA. Six patients with incomplete responses to UDCA were recruited in an open-label study (239). Patients were given 2 doses of rituximab separated by 2 weeks and followed for 52 weeks. This study showed a significant reduction in serum AMA titres and a reduction in ALP up to 36 weeks after treatment. A subsequent open-label study using the same method of treatment enrolled 14 patients with PBC refractory to UDCA (240). B cells were effectively depleted in 13 of the patients, and a reduction in serum AMA levels was observed at 6 months follow-up. However, the improvements in liver biochemistry were limited. Rituximab has also been used in a randomised trial of 57 PBC patients suffering with severe fatigue (241). Despite evidence to suggest that rituximab was effective for reduction of fatigue in a number of conditions including primary sjogrens syndrome (pSS) (242–245), a condition associated with PBC, this study showed no evidence of effectiveness for the treatment of fatigue in PBC.

Although these studies showed the limited efficacy of rituximab in PBC, they demonstrated that the drug is well tolerated by patients. This is in direct contrast to a study with a xenobiotic induced murine model of human PBC (246), where anti-CD20 treatment exacerbated liver pathology despite successful depletion of B cells and reduction in the production of AMAs (247). Conversely, in the genetic animal model of PBC, the dnTGF-βRII mouse (248), anti-CD20 treatment was effective at attenuating liver damage but exacerbates colitis (249). Moreover, this reduction in liver inflammation was only seen in young mice, as B cells depletion in old mice did not modify the course of liver disease (249). Interestingly, double transgenic mice with PBC and B cell depletion (lgμ^-/-^dnTGF-βRII mice) developed a more severe form of cholangitis (250), suggesting that during the initial inflammatory response in this model of PBC, B cells have a suppressive effect.

### Rituximab in Primary Sclerosing Cholangitis

A lack of understanding of PSC pathogenesis has prevented the development of effective therapies. Transplantation was established as the only curative treatment option for PSC in 1983. A few years later, recurrence after liver transplantation was noted in some patients (251). It is estimated that recurrent PSC occurs in 20-25% of patients over a 10-year period after transplantation (252). A small study of 5 PSC patients who underwent ABO incompatible liver transplantation and were treated with rituximab, found that graft survival rate was 100% with no cases of recurrence over the median follow-up period of 7.2 years (253).

## Conclusion

Despite advances in liver T cell biology, B cell biology and subset characterisation remains understudied in the context of chronic liver disease. Deep phenotyping approaches such as single cell RNA sequencing and spatial transcriptomics have yielded valuable information on liver immunity in the context of various liver cell types (254), and similar approaches are much needed for B cell biology. Mapping the B cell compartment in liver diseases will provide a better understanding of the roles of B cells in disease progression and offer new opportunities for therapeutic intervention.

## Author Contributions

AP, YL, DS-T, and ZS researched and composed the review. SD, RB, DK, and GR provided helpful critique of the manuscript. GMR and ZS are joint last authors. All authors contributed to the article and approved the submitted version.

## Funding

This work is supported by a Birmingham Women’s and Children’s Hospital Charity BCHRF546 to AP, a NC3R trainee postdoctoral fellowship UKRI NC/R002061/1 to SD, a Medical Research Foundation intermediate career fellowship UKRI, MRF-169-0001-F-STAM-C0826 to ZS, an EU/EFPIA Innovative Medicines Initiative (IMI) 2 RTCure 777357 to DS-T and a Wellcome Trust funded PhD programme “MIDAS” 108871/B/15/Z to YL and DS-T.

## Conflict of Interest

The authors declare that the research was conducted in the absence of any commercial or financial relationships that could be construed as a potential conflict of interest.

## Publisher’s Note

All claims expressed in this article are solely those of the authors and do not necessarily represent those of their affiliated organizations, or those of the publisher, the editors and the reviewers. Any product that may be evaluated in this article, or claim that may be made by its manufacturer, is not guaranteed or endorsed by the publisher.
